# A stokes polarimetric light microscopy view of liquid crystal droplets

**DOI:** 10.1038/s41598-021-95674-4

**Published:** 2021-08-11

**Authors:** J. Gou, T. H. Shen, P. Bao, J. L. Ramos Angulo, S. D. Evans

**Affiliations:** 1 Joule Physics Laboratory, School of Science, Engineering and Environment, University of Salford, Newton Building, Salford, M5 4WT UK; 2 Optimum Imaging Ltd, Sixth Floor, Maxwell Building, Salford, M5 4WT UK; 3 School of Physics and Astronomy, University of Leeds, Leeds, LS2 9JT UK; 4 Present Address: Institute of Modern Physics, Chinese Academy of Sciences, 509 Nanchang Road, Lanzhou, 730000 China

**Keywords:** Materials science, Optics and photonics, Physics

## Abstract

The optical characteristics of materials, such as their magnetooptical effects, birefringence, optical activities, linear and circular dichroism, are probed via the polarisation states of light transmitted through or reflected from the specimens. As such, the measurements of the polarisation states play an important role in many research disciplines. Experimentally, Stokes parameters provide a full description of the polarisation states of light. We report the implementation of a dual- photoelastic modulator based polarimeter in a light microscope, enabling the determination of Stokes parameters at each pixel. As a case study, polarimetric images of liquid crystal droplets of different internal structures are obtained, showing their distinct polarisation characteristics. We demonstrate that the prototype Stokes polarimetric microscope allows the quantitative determination of the polarisation characteristics of light at the object plane and enables the access of the information of full polarisation states as compared to a conventional cross polariser microscope. This work shows that Stokes polarimetric microscopy may find potential applications in a wide range of research fields.

## Introduction

Recent progress in producing mono-disperse liquid crystal (LC) droplets and shells with controlled sizes has generated significant interest in the study of their physical characteristics and the exploration of their potential applications^1^. Cross-polariser light microscopy, i.e., conventional polarisation microscopy, has been widely used in capturing the polarisation ‘textures’ in the study of, for instance, the molecular alignments (the ‘director’ fields) and topological defects, making use of the strong optical anisotropy associated with such systems^2^, whereas simulations have also been available with such polarisation microscopic configuration to help elucidate the observations^3,4^. However, conventional cross- polariser microscopy can only obtain partial information of the polarisation state of light. For instance, when a spherically symmetric droplet is illuminated with a linearly polarised light, one would expect that the polarisation image of the droplet would have shown a two-fold rotational symmetry. Instead, under cross-polariser imaging, the droplet always appears to have a ‘hot cross bun’ shaped four-fold symmetry^5^, having two orthogonal dark lines aligned in the direction of the polarisers which reflects the symmetry imposed by the measurement system.

Broadly speaking, accessing to the polarisation information of light can be achieved through either a combination of a retardation plate and a polariser at various angular settings or with modulators. For the former, by introducing polarisers and retardation plates to a light microscope, such a polarised light microscope enables a wealth of applications in materials research, for instance in micrometry, crystal morphology and dispersion staining (a particle identification technique based on the difference between the refractive index of a particle and that of the liquid medium in which the particle is immersed)^6^ For the latter, variable liquid crystal retarders have been deployed in Mueller matrix polarisation microscope^7^ and in ‘PolScope’ which is usually calibrated for birefringence measurements^8^.

Quantitative polarisation measurements, i.e., polarimetry, have a long history in scientific research, for instance, in astronomy^9–11^ in remote sensing^12^, in magnetooptical studies^13^ and in the context of ellipsometry^14^ and Mueller matrix determination^15–17^. Furthermore, photoelastic modulator (PEM) based polarimetry has recently found its application in plasma physics, where the polarisation of helium atomic emission was used to probe the electron velocity distribution in electron cyclotron resonance heating^18^. Polarimetry has also broad industrial applications, for measuring the concentration of optically active substances in a solution. Such polarimeters are used extensively in the food and pharmaceutical industries^19^.

Stokes parameters^20^, describing the full state of the polarisation of light, are: the intensity, *I*; the intensity difference of the linear polarised components along two orthogonal directions, *Q*, of a chosen laboratory coordinate system; the intensity difference of the linear polarised components, *U*, along two orthogonal directions but rotated by 45° with respect to that in *Q*; and the intensity difference, *V*, of the left and right-handed circular polarisation components. Knowing Stokes parameters enables the determination of any polarisation characteristics of light, such as polarisation azimuthal angle, ellipticity angle and percentage of polarisation for partially polarised light. Usually, to measure the Stokes parameters, four different polarisation optical arrangements are required. Several methods have been proposed to measure the Stokes parameters, for instance, with a rotating retarder^21,22^, by the division-of-amplitude^23^ and, in Stokes polarimetric imaging, with the division-of-aperture using Wollaston prism array^24^, the division-of-focal-plane with an array of groups of micro polarisers and retarders in front of the digital sensor^25–27^ and with matrix Fourier optics^28^.

The use of optical phase modulators has enabled the measurement of the four parameters in a single optical setting, of which the photoelastic modulators provide a fast and accurate optical phase modulation and, with a typical fundamental modulation frequency of several tens of kilohertz, highly preferable for the deployment of lock-in amplifiers for sensitive signal recovery. A dual-PEM based polarimeter is made of two PEMs and an analyser^29^. The phase modulations ‘fold’ the signals associated with the original Stokes parameters to the coefficients of the DC and different harmonics of the modulations in the output intensity, which can be recovered separately after the analyser, using lock-in signal detection techniques. Guan et al. have described the dual-PEM based polarimeter and the calibration methods to enable the absolute measurements of the polarisation states [ref^30^, and the references therein,] and a non-linear optimization method has been employed to determine the calibration constants for the polarimeter^31^.

To apply PEM based polarimetry to imaging, it is necessary to operate the lock-in technique at the individual pixel level. Unlike the approach where specially masked CCD detector with three out of four rows covered for the demodulation of the signals^11,32^, in this work we report a dual PEM polarimeter based prototype of Stokes polarimetric microscope, implemented using a commercially available digital camera in conjunction with a purpose-built demodulation timing unit, enabling the lock-in signal recovery at each pixel. For the Mueller matrix polarisation microscope and the PolScope, using variable liquid crystal retarders, the measurements are taken at selected retarder settings to allow the extraction of the Mueller matrix or the birefringence data, which is essentially ‘DC’ level measurements. As such, it is necessary that the background intensity variation is suitably corrected^7,8^ and the measurements are vulnerable to intensity fluctuations. In contrast, dual- PEM polarimetry is based on the lock-in technique, so it is less vulnerable to background intensity variations. Polarisation characteristics depends on the ‘normalised’ Stokes parameters *q* = *Q*/*I*, *u* = *U*/*I* and *v* = *V*/*I*, which are independent of the background intensity variations. Also for imaging of purely polarisation characteristics, since the Stokes parameter of the intensity of purely polarised light, ${I}_{p}$, can be written as^33^ ${{I}_{p}}^{2}={Q}^{2}+{U}^{2}+{V}^{2}$, the measurement of the DC component of the signal may even be entirely avoided in this case.

For the present work, we focus on the imaging of Stokes parameters and the subsequent determination of the polarisation characteristics such as polarisation azimuthal angle and ellipticity angle images. For applications requiring the determination of the Mueller matrix elements of the specimen, a minimum of four sets of Stokes parameters images will be required with different known input polarisation states of linear and circular polarisation^15^. If the specimens are primarily uniaxial birefringent, the orientation of the crystal optical axis and the retardation may in principle be determined at each pixel from a single set of Stoke parameters with judiciously chosen elliptical polarised light illumination. (Please refer to Supplementary Materials for further details.) For more complex systems, general methods^34,35^ developed for the extraction of effective parameters of anisotropic optical materials are applicable to the Stokes parameter imaging.

We demonstrate the application of the prototype PEM based Stokes polarimetric microscope to the polarimetric imaging of liquid crystal droplets which have distinctly different internal structures.

## Materials and methods

Monodisperse liquid crystal (LC) droplets were produced using a microfluidic device, as described in the previous work^36^. Commercial nematic LC mixture ‘E7’, containing 51% 4-Cyano-4'-pentylbiphenyl (5CB), 25% 4-cyano-4'-n-heptyl-biphenyl (7CB), 16% 4-cyano-4'-n-oxyoctyl-biphenyl (8OCB) and 8% 4-cyano-4''-n-pentyl-terphenyl (5CT), is used for the production of the LC droplets. Two types of coating layers are used: one is a monolayer of the mixture of lipids 1,2-dioleoyl-sn-glycero-3-phosphocholine (DOPC) and 1,2-dioleoyl-sn-glycero-3-phospho-rac-(1-glycerol) sodium salt (DOPG) and the other polyvinyl alcohol (PVA). We shall present the polarimetric images of LC droplets with three distinct internal structures: (A) DOPC/DOPG (1:1) coated E7 droplets (heated to 70 °C, held for 20 min then cooled to room temperature), in which the liquid crystal molecules are radially aligned, with a topological defect at the centre of each droplet; (B) PVA wrapped E7 LC droplets, in which the molecules have a planar anchor at the interface, resulting in a bipolar symmetry for each droplet and two topological defects at the opposite poles on its surface; and (C) droplets of PVA wrapped E7 doped with the chiral nematic liquid crystal twist agent ‘Merck S1011’, with planar anchoring of the molecules at the surface of the droplets and a helical ordering along the radial direction with a topological defect at the centre of the droplets.

The schematic diagram of the prototype Stokes polarimetric microscope is shown in the Supplementary Materials. In this work, the polarimetric imaging has been implemented as follows. The two PEMs (Hinds Instruments) of the polarimeter have their optical axes aligned approximately 45° to each other and the polarising analyser with its passing axis bisecting the angle at approximately 22.5°. We have incorporated the dual-PEM based polarimeter in an Olympus IX-71 ‘inverted’ light microscope for biological applications. The polarimeter is installed in the ‘infinity space’ of the microscope, between the objective lens and the tube lens where the light pencil from a point on the object would be parallel. This minimises any impact of the insertion of the polarimeter to the imaging optics. The parallel beams also ensure uniform retardation as the light beams traverse the PEMs. The light pencil originated from different points of an object will traverse parallelly through the PEMs, but at different angles, thereby experience a slightly different retardation. In principle, the effect of the difference could be corrected if the calibration is conducted at the pixel level (please referr to the Supplementary Materials for details of the calibration). In practice, the focal length of the tube lens is 180 mm and the camera sensor diagonal length 22 mm. This represents an angle of *ζ* =  ± 3.5° from normal incidence for the pencil of light collected at the corner pixels, with a correction to peak retardation of 1/cos(*ζ*) representing a less than 0.2% increase in the peak retardation, which is negligible in the present work.

A purpose-built electronic ‘demodulation timing unit’ (DTU) enables a commercially available digital camera to be used for phase lock-in signal recovery at the pixel level, so that no specialised development on camera chipset is required and the performance may be improved with better commercial cameras in future. The DTU takes input from the PEMs reference frequency and generate a phase-locked square wave output with a mark-space ratio of 1:1 at either the fundamental frequency of the PEMs or their second harmonics. The DTU output is used to control the switching of the microscope illumination source, which consists of a PicaQuant picosecond pulsed laser operated at 80 MHz repetition rate, model ‘LDH-P-C-670’, 670 nm wavelength, coupled via an optical fibre to the Koehler illumination source optics. An Optotune laser speckle reducer, model LSR-3005 -24D_VIS, operated at 300 Hz, is used in the source optics to remove laser speckles. The DTU has a timing accuracy of about 6 ns to ensure a timing precision to single pulse of the laser. The DTU driven illumination source would allow the light signal integration of only the chosen half-cycle whilst the sensor at each pixel acts as a signal integrator of the same half-cycle over a given number of cycles of the modulation. An Andor Zyla 5.5 camera (2560 × 2160 pixels) is used as the imaging pixel array. A 180° phase-shifted DTU output allows the signal integration of the other half-cycle to form a pair of measurements of a given frequency. The difference between the two is proportional to the signal associated with that frequency and the sum proportional to the average (DC) component. This is similar to the working principle of a conventional lock-in amplifier^37^, which rejects higher even harmonics of the phase locked frequency, whilst for the PEMs methodology^30^ additional filtering of higher odd harmonics is not required. A 90° phase shift of the DTU output for the pair of measurements permits the measurement of the signal at the quadrature to the reference. The image acquisition can be repeated to extend the dynamic range of the signal detector. The determination of the Stokes parameters requires the measurement of the DC signal *S*_DC_, the signals associated with the 1f. frequency, *S*_V_, and the 2f. frequency, *S*_QU1_, of the first PEM modulation and that associated with the 2f. frequency, *S*_QU2_, of the second PEM modulation. To obtain a full measurement of the Stokes parameters, twelve measurements are taken sequentially using the prototype microscope. The Stokes parameters, *I*, *Q*, *U* and *V*, are related to *S*_DC_, *S*_QU1_, *S*_QU2_ and *S*_V_ by a 4 × 4 matrix, *K*, of the following form:

1 $$K = \begin{array}{*{20}c} {\left( {\begin{array}{*{20}c} 1 & {k_{1} } & {k_{2} } & 0 \\ 0 & {k_{3} } & {k_{4} } & 0 \\ 0 & {k_{5} } & {k_{6} } & 0 \\ 0 & 0 & 0 & {k_{7} } \\ \end{array} } \right),} & {} \\ \end{array}$$

where *k*_1_…*k*_7_ are the non-zero elements related to the angular setting of the polarimeter and the peak retardation values of the two PEMs. The non-zero elements of *K* can be determined via a calibration procedure of the polarimeter^30,31^ using a range of input polarisation states. This method has also the advantage that the precise values of the pre-set peak retardations of the dual-PEM do not need to be known.

## Results

For a description of the experimental setup, please refer to the ‘Materials and methods’ section above and the Supplementary Materials.

**Figure 1:**
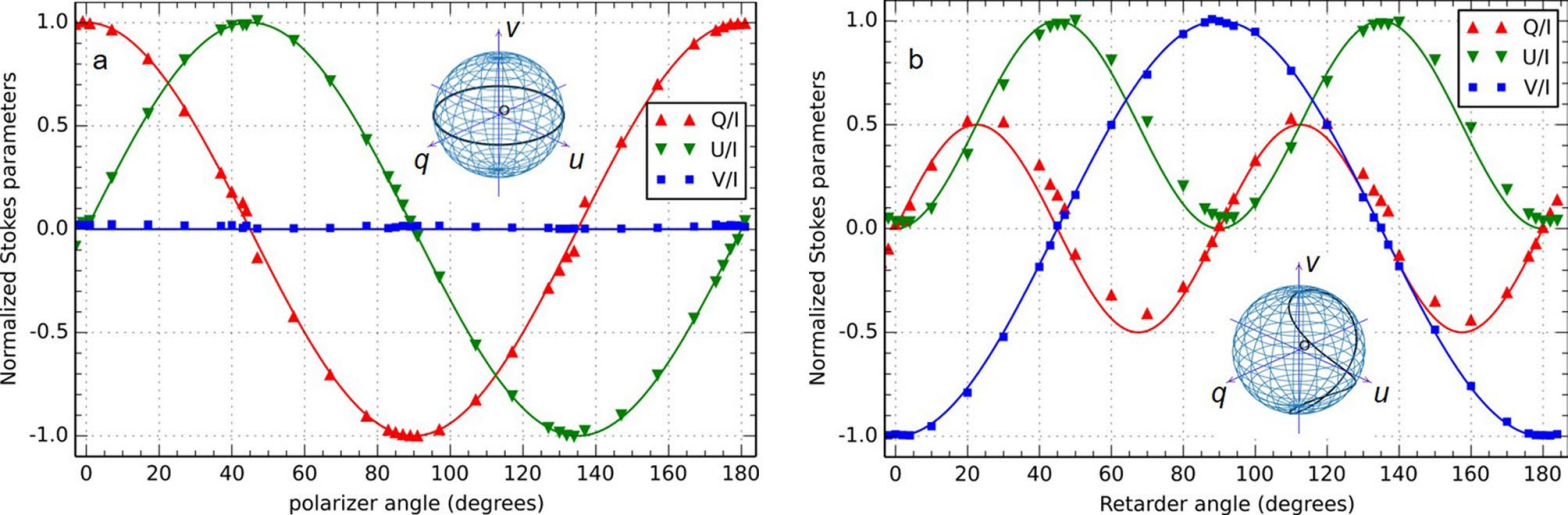
Data showing the normalised Stokes parameters with polarisation states generated for determining the non-zero elements of the matrix *K* using a non-linear optimisation calibration procedure^5^. (**a**) shows polarisation states generated along the equatorial great circle of the unit Poincaré sphere (inset in (**a**)), whilst (**b**) the elliptical states along the curve indicated on the surface of the sphere (inset in (**b**)). Both data are combined for the calibration. The lines indicate the expected theoretical behaviour.

Figure 1 shows the result of the calibration of the polarimeter. For practical convenience, the calibration was carried out with the objective lens removed, using the source, collimated, directly in front of the polarimeter and the signals were collected near the centre of the image. Since the image plane is the focal plane of the tube lens, the light collected would have been parallel in the polarimeter, just the same as when the objective lens was in place. The effect of the polarisation preserving objective lens was found to be very small and no numerical correction was necessary for this work. Different polarisation states are generated before the light entered the polarimeter. Figure 1a shows, with the ‘normalised’ Stokes parameters, the calibration curves when a linear polariser is placed in front of the polarimeter and is rotated to generate linearly polarised light with different azimuthal angles. The solid lines are the theoretical curves, whilst the points are the experimental measurements from which the non-zero elements in the matrix *K* are determined. The inset shows the polarisation states thus sampled on the unit Poincaré sphere, which is along the equatorial great circle as indicated. Figure 1b shows the data obtained with a combination of a linear polariser and a quarter waveplate to generate different states of elliptically polarised light. To determine the value *k*_7_, it is necessary to generate elliptical and circular polarisation states for the calibration process. The algorithm requires several different polarisation states with large circular polarisation components, but precise knowledge of the states is not required. These polarisation states are generated by the combination of a polariser and quarter wave plate, with the retarder rotated with respect to the polariser. For polariser azimuth angle at 45° and the retarder rotated from 0 to 180°, the polarisation states generated are along the curve on the surface of the unit Poincaré sphere depicted in the inset of Fig. 1b, corresponding to the curves shown in Fig. 1b. It is important to include the data points of strong circular polarisation components (near the polar region on the sphere) in the calibration process to obtain reliable value for the element *k*_7_ of the calibration matrix. The calibration also provides a useful diagnostic tool for the assessment of the polarimeter performance, for instance, for purely linear polarisation input, any non-zero *V* value would indicate possible ‘cross-talk’ between different modulation frequencies, which has been found negligible in the present experimental setup. For the observation of the polarimetric images of LC droplets, a linear polariser is placed between the illumination source and the specimen. All images are obtained in transmission using the polarimetric microscope.

**Figure 2:**
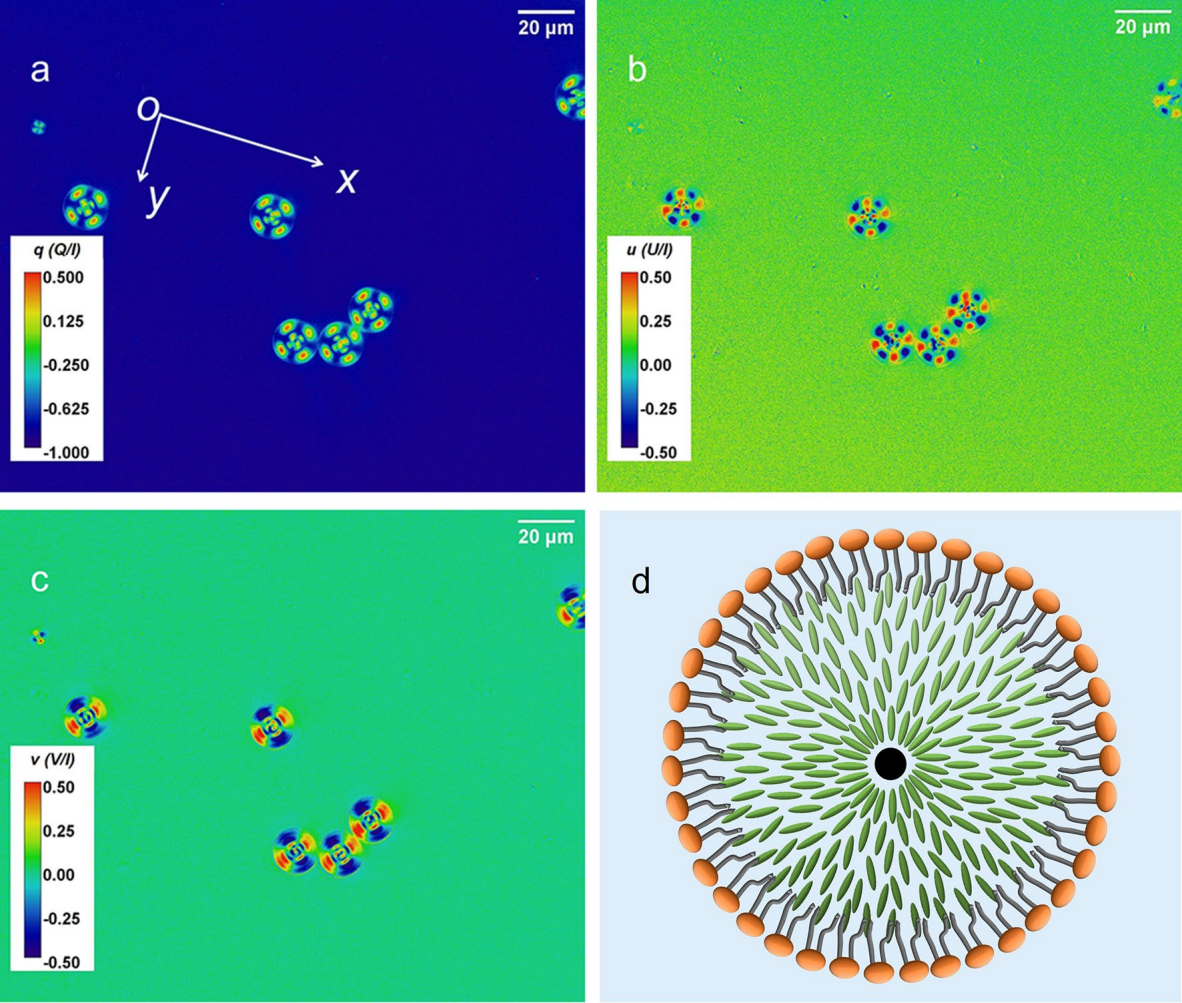
Images of normalised Stokes parameters of (**a**) *q*, (**b**), *u* and (**c**) *v.* for LC droplets of structure A. (**d**) A schematic drawing (not to scale) of the internal structure of the LC droplets, depicting a monolayer of lipids mixture (DOPC/DOPG) on the surface of the droplet. The *x*-axis indicates the direction of the optical axis of the 2nd PEM, which defines the laboratory coordinates for the polarisation states measured. For this measurement, the polarisation plane of the incident linear polarised light is approximately along the *y* -axis.

The polarimetric images of the mono-disperse LC droplets of E7 wrapped by a DOPC/DOPG lipid layer, the structure A, are shown in Fig. 2. Figure 2a–c are the images of the normalised Stokes parameters *q*, *u*, and *v*. The schematic diagram in Fig. 2d illustrates (not to scale) the internal structure of the droplets with the structure A. The 2nd PEM optical axis defines the laboratory coordinates of the polarimeter^30^, which is indicated by the *x*-axis in Fig. 2a, about 16° to the horizontal edge of the image for the experimental setup. Since *q* and *u* are the normalised intensity difference of the linear polarisation components in two orthogonal directions, and the polarisation plane of the linear polarised incident light is along the *y* -direction, in the background of Fig. 2a *q* is equal to − 1, and, in the background of Fig. 2b, *u* is 0. As the LC molecules have a radial alignment in the droplets, being spherically symmetrical, all droplets display the same overall polarisation characteristics. The *q* and *u* images show the droplets having a four-fold rotational symmetry, like those in the cross-polariser image (please refer to Fig S2b) in the Supplementary Materials, where conventional transmission and cross-polariser light microscopic images are shown for all three types of the LC droplets). Figure 2c shows the image of *v,* the normalised intensity difference of the left-hand and the right-hand circular polarisation components. As the incident light is linearly polarised, *v* in the background of the image is approximately equal to zero. Interestingly, this image reveals a two-fold rotational symmetry of the droplets, as expected, with linear polarised incident light. The droplets have caused significant changes in all three normalised Stokes parameters.

**Figure 3:**
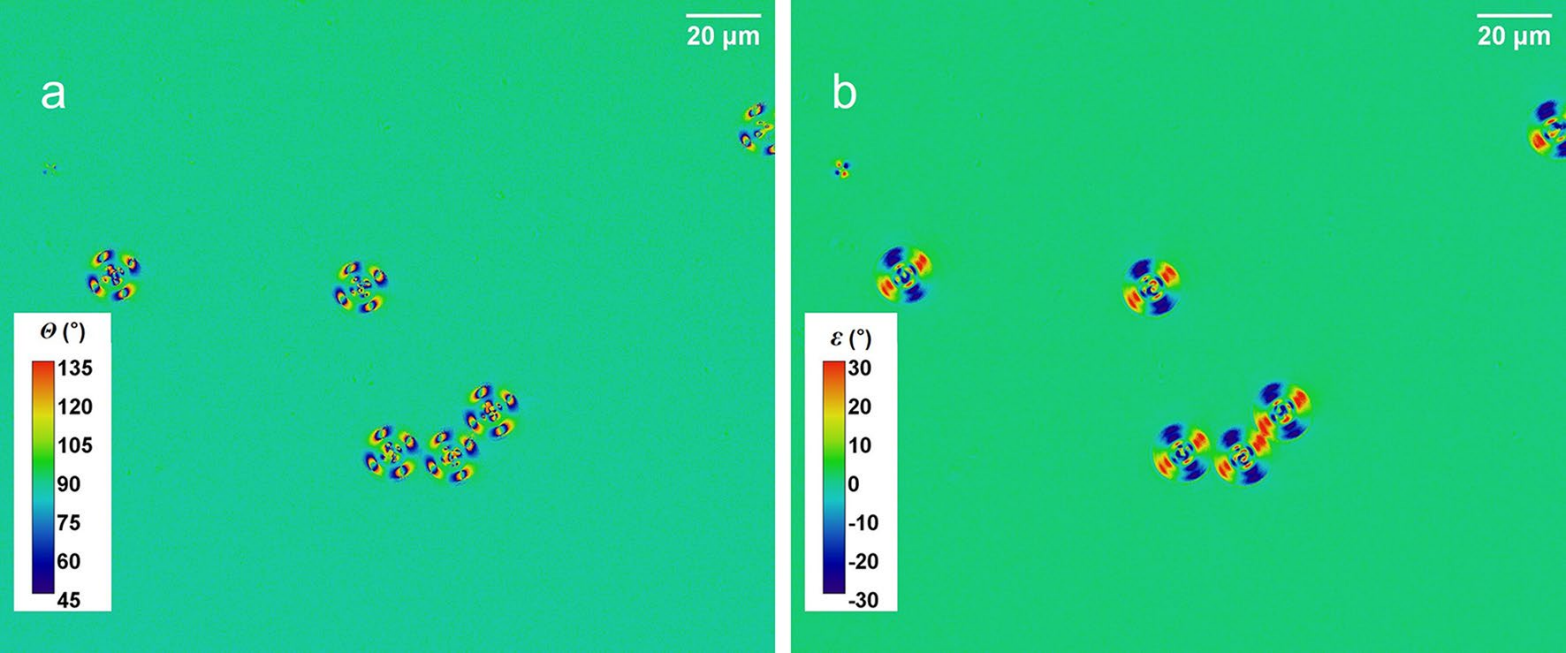
The images show (**a**) the polarisation azimuthal angle, *θ*, and (**b**) the ellipticity angle, *ε*, in the same field of view as that in Fig. 2.

Stokes parameters enable the evaluation of any polarisation characteristics such as polarisation azimuthal angle, *θ*, and ellipticity angle, *ε*. Figure 3a shows the image of the polarisation azimuthal angle, *θ*, with respect to that of the incident light, again displaying a four-fold rotational symmetry pattern of the LC droplets. In contract, in Fig. 3b the image of ellipticity angle, *ε*, is shown to have a two-fold rotational symmetry of the LC droplets. The image shows that the interaction of the linear polarised light interacting with the spherical droplets has led to the changes of ellipticity angle in the opposite sense of handedness in two orthogonal directions, thereby losing the fourfold symmetry observable in the image of polarisation azimuthal angle.

**Figure 4:**
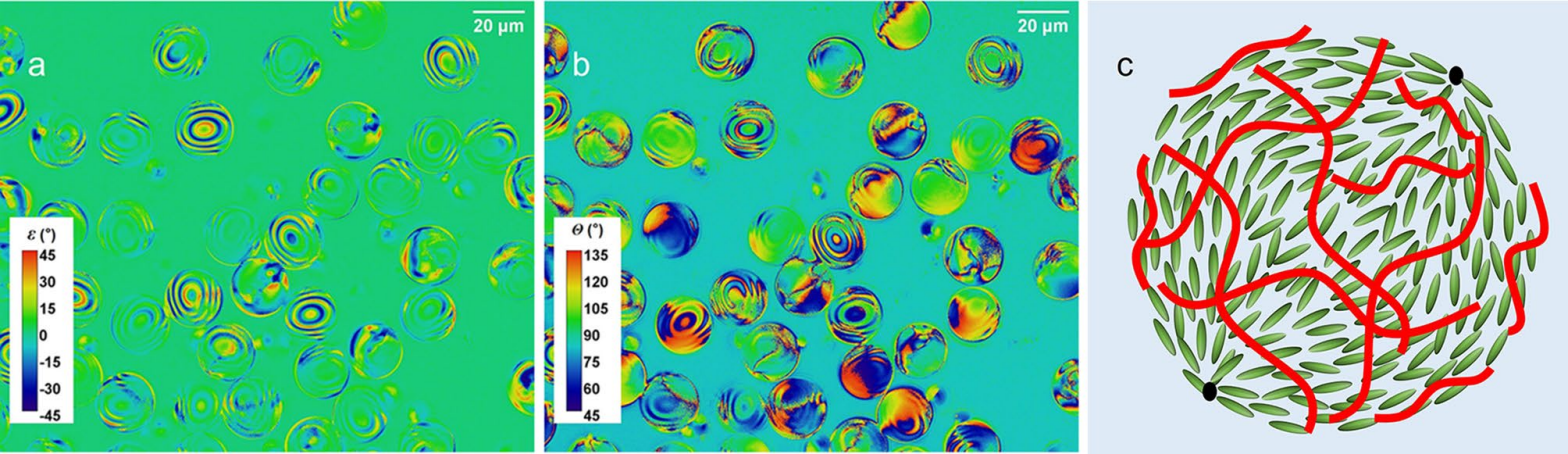
Polarimetric images of LC droplets of structure B, showing (**a**) the ellipticity angle, *ε*, and (**b**) the polarisation azimuthal angle, *θ*. (**c**) A schematic drawing (not to scale) illustrates the internal structure of the LC droplets with a randomly orientated surface layer of PVA molecules represented by red lines.

The polarimetric characteristics of the PVA wrapped E7 LC droplets (the structure B) contrast significantly to that of the structure A. Figure 4a and b show, respectively, the image of the ellipticity angle, *ε*, and the image of the polarisation azimuthal angle, *θ*. Figure 4c shows, not to scale, the schematic structure of the droplets, with the LC molecules in a bipolar alignment. The droplets are known to have a bipolar symmetry with the coverage of a layer of randomly orientated PVA at the droplet surface. This has resulted in different appearances of individual droplets, which would largely be accounted for by the orientation of the axis of the bipolar symmetry. Those with their symmetry axes orientated along the direction of the observation show a degree of circular symmetry surrounding the pole.

**Figure 5:**
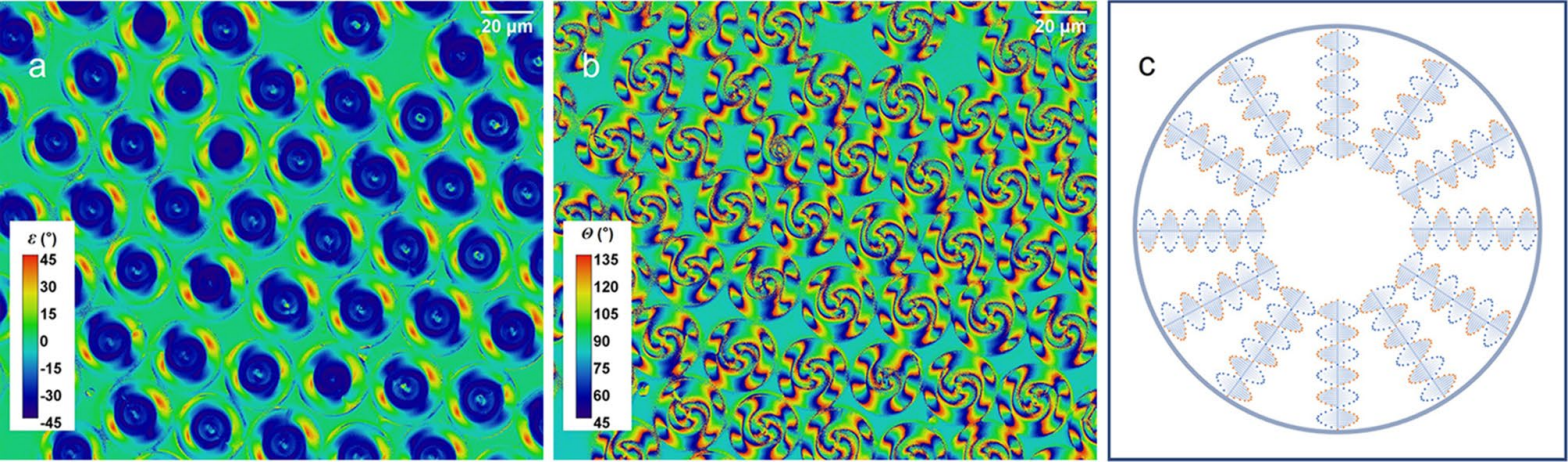
Polarimetric images of LC droplets of structure C, the radially aligned chiral structure, showing (**a**) the image of the ellipticity angle, *ε*, and (**b**) the image of the polarisation azimuthal angle, *θ*. The presence of an almost circular polarisation emerging from a large area of a droplet is clearly visible w**i**th an ellipticity angle of the value close to − 45°. (**c**) A schematic illustration (not to scale) of the internal structure of the LC droplets depicting the spiral structure aligned along the radial direction.

The Merck S1011 twist agent doped E7 droplets with PVA wrapping (structure C) have helical ordering along their radial direction. As shown in Fig. 5, all the droplets look identical in both the image (Fig. 5a) of the ellipticity, *ε*, and the image (Fig. 5b) of the polarisation azimuthal angle, *θ*, which is similar to that of the droplets with the structure A, as both have spherical symmetry. (The schematic diagram in Fig. 5c depicts, not to scale, the LC molecules aligned in a spiral along the radial directions). Also, similarly, the ellipticity image is characterised by a two-fold rotational symmetry and the polarisation azimuthal angle image a four-fold rotational symmetry. Furthermore, the polarimetric ellipticity image shows that a large region on the droplets has an ellipticity angle close to a value of − 45°, indicating a predominant righthanded circular polarisation (with respect to our laboratory coordinates), a consequence of the chiral nematic ordering of E7 in the droplet, which appears to be dominating the polarisation characteristics.

**Figure 6:**
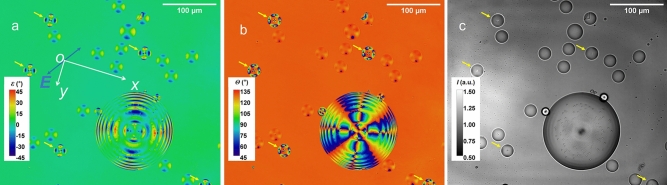
Preliminary results of polarimetric images using a 660 nm LED source, showing the images of (**a**) the ellipticity angle, *ε*, (**b**) the polarisation azimuthal angle, *θ*, and (**c**) the intensity, *I*, respectively. The intensity grey scale in (**c**) is in an arbitrary unit (a.u.). The blue line with a label *E* indicates the polarisation azimuthal angle of the incident linear polarised light. The five yellow arrows identify the droplets happened to be in the same focal plane.

Figure 6 shows the images of LC droplets of the structure A from our preliminary work using an LED source with light emission centred at 660 nm and a 10 nm full-width-half-maximum bandpass filter also centred at 660 nm, in an implementation of the microscope analogous to that described previously, with an accidentally formed ‘over-sized’ droplet in the view. The over-sized droplet was possibly formed due to coalescing smaller droplets during sample transport. Regardless of the differences in size, the droplets all show a two-fold rotational symmetry in the ellipticity image (Fig. 6a) and a four-fold rotational symmetry for the polarisation azimuthal image (Fig. 6b), reflecting the radial alignment of the liquid crystal molecules. Whilst in the normal intensity image (Fig. 6c), all the droplets appeared to be reasonably well in focus, the polarimetric images showed different polarisation contrast (the higher polarisation contrast droplets are indicated by a yellow arrow, which happened to be in the same focal plane). Such relative contrast difference could be changed as the focal position is slightly adjusted, suggesting that the polarisation characteristics are sensitive to focusing. This feature may merit further investigation, and could be potentially useful in improving depth sensitivity in future, for instance, in a confocal microscope implementation of the polarimetry technique.

## Discussion

In comparison with the cross-polariser microscope images (please refer to Supplementary Materials), the Stokes polarimetric microscope images provide more quantitative information regarding the polarisation states of light at the object plane. They show that the LC droplets cause both changes to the plane of polarisation of light and its ellipticity. Those changes are separately quantified. The effect of chiral molecular ordering can also be quantified in the ellipticity angle imaging. To make full use of the experimental observations, relevant theoretical modelling of the interaction of light and LC droplets would be necessary to elucidate the experimental observations.

Our results show that the two-fold rotational symmetry associated with a linearly polarised light interacting with a droplet of spherical symmetry is observable and only associated with a phase change that lead to non-zero ellipticity angle. In the absence of precise theoretical modelling, we shall try to briefly explore the possible causes within the context of existing literature.

Polarisation state may change at a boundary between two media as described by the Fresnel equations for the amplitude transmission and reflection coefficients^33^. For completely non-absorbing media, the coefficients are real, so whilst they may cause the changes in the orientation of the polarisation plane of linearly polarised light, they do not change the linear polarisation into an elliptical polarisation. To introduce a finite ellipticity angle, the coefficients will need to be complex, which may exist if the droplet is absorbing, resulting in complex refraction indices. However, considering that the polarimetric images were taken well below the absorption edge of E7 liquid crystal mixture^38^, a direct application of Fresnel’s equations is unlikely to yield the observed behaviour. On the other hand, the response of a homogeneous, isotropic, dielectric sphere in a homogenous, uniform electric field is of the form of a dipolar field^39^. Studies on nano-spheres in Mie scattering have been reported^40,41^, whereas recent work on the emission of circularly polarised light by a linear dipole^42^ would suggest a possible route in establishing a theoretical model for the experimental observations. We note that LC droplets are effectively micro-lenses with their internal structures drastically affecting the polarisation characteristics as light interacting with them. Therefore, a comprehensive model would need to consider the effects of the boundaries, the appropriate optical response functions of the liquid crystals and the light wave propagation through the droplets. Full numerical modelling in solving Maxwell’s equations with appropriate boundary conditions may provide further insight, taking into consideration the geometric factors, the materials characteristics and the wave propagation.

To summarise, a Stokes polarimetric light microscope, based on dual-PEM polarimeter methodologies, has been described. With the aid of the prototype microscope, we have presented a polarimetric view of E7 liquid crystal droplets of contrasting structures. The prototype microscope is able to provide quantitative measurements of the Stokes parameters at each pixel. The polarimetric images of LC droplets suggest that their internal structure may drastically affect the polarisation states of light traversing through the droplets. We have observed a twofold symmetry in ellipticity images for droplets of radial symmetry, which was not observable by conventional cross-polariser light microscopy and speculated its origin. Whilst the present work focuses on the Stokes parameters, the microscope is applicable to Mueller matrix and birefringence imaging. We believe the work may stimulate numerical modelling of polarised light interaction with novel materials and mesoscopic systems and the technique of Stokes polarimetric imaging we have proposed may find its applications in a wide range of disciplines.

## Supplementary Information

Supplementary Information.

## Data Availability

All raw data related to this work are available upon email request to the corresponding author.
