# Supplementary material for: A stokes polarimetric light microscopy view of liquid crystal droplets
Source: Sci Rep. 2021 Aug 11;11:16329. doi: 10.1038/s41598-021-95674-4 (PMC8358033; doi:10.1038/s41598-021-95674-4)
Supplement: Supplementary file 1 — Supplementary Information. [file 41598_2021_95674_MOESM1_ESM.docx]

Title: A Stokes Polarimetric Light Microscopy View of Liquid Crystal Droplets

Authors: J Gou^1^, T H Shen^1,2*^, P Bao^3^, J L Ramos Angulo^2^, S D Evans^3^

^1^Joule Physics Laboratory, School of Science, Engineering and Environment, University of Salford, Newton Building, Salford, M5 4WT, U.K.

^2^Optimum Imaging Ltd, Sixth Floor, Maxwell Building, Salford, M5 4WT, U.K.

^3^School of Physics and Astronomy, University of Leeds, Leeds LS2 9JT, U.K.

Contact details:

Jie Gou, Joule Physics Laboratory, School of SEE, University of Salford, Newton Building, Salford, M5 4WT, U.K.; email: [j.gou@edu.salford.ac.uk](mailto:j.gou@edu.salford.ac.uk); current address: Institute of Modern Physics, Chinese Academy of Sciences, 509 Nanchang Road, Lanzhou, China, 730000; email: goujie@impcas.ac.cn

Tiehan H Shen (corresponding author), Joule Physics Laboratory, School of SEE, University of Salford, Newton Building, Salford, M5 4WT, U.K. and Optimum Imaging Ltd, Sixth Floor, Maxwell Building, Salford, M5 4WT, U.K.; email: [t.shen@salford.ac.uk](mailto:t.shen@salford.ac.uk); tel: +44 161 2952544

Peng Bao, School of Physics and Astronomy, University of Leeds, Leeds LS2 9JT, U.K.; email: [p.bao@leeds.ac.uk](mailto:p.bao@leeds.ac.uk)

Jorge L Ramos Angulo, Optimum Imaging Ltd, Sixth Floor, Maxwell Building, Salford, M5 4WT, U.K.; email: [jorgeramos2591@gmail.com](mailto:jorgeramos2591@gmail.com)

Stephen D. Evans, School of Physics and Astronomy, University of Leeds, Leeds LS2 9JT, U.K.; email: s.d.evans@leeds.ac.uk

**Supplementary Materials**

The supplementary materials contain the following: a description and the schematic diagram of the prototype dual - PEM based Stokes polarimetric microscope, the methodology for the calibration of the polarimeter and the relationship between Stokes polarimetric imaging, Mueller matrix imaging and birefringence imaging. Also included are the transmission light microscopy intensity images and cross-polariser polarisation microscopy images of the LC droplets, which may be compared to the polarimetric images presented in the Article.

**The optical setup of the prototype dual-PEM Stokes polarimetric microscope**

The prototype polarimetric microscope was built on the modifications to an existing Olympus IX 71 light microscope for biological applications. A schematic diagram of the optical setup is shown in Figure S1(a).


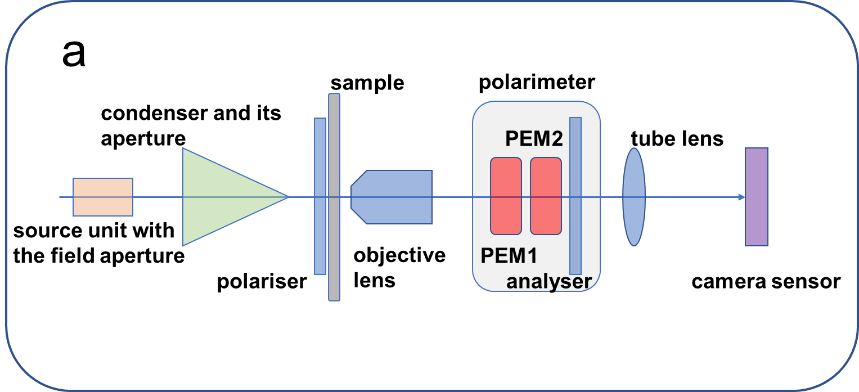

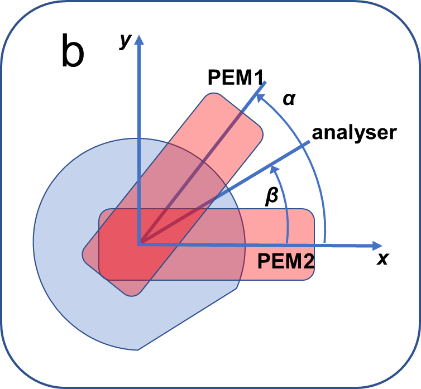


Figure S1. (a) Schematic diagram showing the components of the dual-PEM based Stokes polarimetric microscopy. (b) Viewed along the direction of light propagation, the polarimeter setting defines the laboratory coordinates, in which the fast axis of PEM2 is the *x*- axis, the angle between the fast axis of PEM1 and the *x*-axis is *α* and the angle between the passing axis of the analyser and the *x*-axis is *β*.

When using the 670 nm PicaQuant picosecond pulsed laser (model LDH-P-C-670) as the light source, the laser outputs to an optical fibre, which is coupled to an aspherical lens followed by an Optotune laser speckle reducer (model LSR-3005 -24D_VIS). This replaces the original halogen source. For the preliminary work using light emitting diode (LED), a Thorlabs 660nm collimated LED for Olympus IX microscope (model M660L4-C1) replaces the original source, with a bandpass filter of 10nm full-width-half-maximum and centred at 660nm inserted in the filter slot of the source unit. The adjustment of the source and the condenser unit allows a choice of either Köhler or critical illumination. In the present work, only Köhler illumination is used.

Polarisation generating optics may be placed in front of the sample. For our present work, it is simply a linear polariser. Polarisation preserving objective lenses are used for the imaging.

The dual-PEM based polarimeter is inserted in the ‘infinity space’ of the microscope, between the objective lens and the tube lens. This is the region where a pencil of light from a point of the sample will be parallel, experiencing the same retardation through the PEMs. Pencils from different points of the sample will be parallel themselves but at different angles to each other. However as those points will be refocused by the tube lens at different pixels on the image plane, in principle, when the calibration is conducted at pixel level, the difference in the retardations for different pencils of light may be corrected (please refer to the calibration methodology later). As discussed in the Article, the maximum angular difference is determined by the focal lens of the tube lens and the size of the camera sensor. The correction would amount to a maximum of about 0.2% in terms of the peak retardation of the PEMs, which was found to be unnecessary for the present work.

For practical convenience, the calibrations were conducted with the collimated source being placed directly in front of the polarimeter. The objective lens is expected to introduce an additional polarimetric background. However, for the present work, the objective lenses used have been found to have negligible effect on the background polarisation states and no numerical correction was required for the LC droplets imaging.

An Andor CMOS camera (Model Zyla 5.5) is used for the signal detection. The camera sensor has 2560×2160 pixels and a diagonal length of 22 mm. With a 40× objective lens there are about 6 pixels per micron length of an object when the ‘1×’ option for the tube lens is chosen. The sensor has a low gain and a high gain pre-amplifier for each pixel with the actual analogue to digital conversion (ADC) carried out at 12 bits to provide a combined16 bits ‘dynamic range’ across an image (c.f. Zyla sCMOS Hardware Guide). To maintain high sensitivity for all pixels for the lock-in signal recovery, only the option of high gain pre-amplifier with 12 bits ADC is suitable for our applications. The 12 bits ADC provides a dynamic range of 4096 per exposure. Accumulation of multiple exposures may be used to extend the range as the individual frames are added digitally following the exposures. LabVIEW is used for the computerised image acquisition. Polarimetric images are saved in the format of Flexible Imaging Transport System (FITS) to preserve all numerical values for each pixel. For the present work, we use ImageJ for the display of the polarimetric images either in greyscale or in false colour (using the colour look-up table ‘physics’) and the images are exported in the format of Joint Photographic Experts Group (jpeg) for digital portability.

**The calibration of the polarimeter**

Figure S1(b) illustrates schematically the angular configuration of the dual- PEM polarimeter. Light traverses through PEM1, PEM2, the analyser to the detector. The fast axis of PEM2 is the *x*- axis. The angle between the fast axis of PEM1 and the *x*- axis is *α*. The angle between the passing axis of the analyser and the *x*-axis is *β*. The fundamental frequency of PEM1 is $\Omega_{1}$ and that of PEM2 is$\Omega_{2}$. The peak retardations of PEM1 and PEM2 are *δ*_10_ and *δ*_20_ respectively. Let *I*, *Q*, *U* and *V* denote the Stokes parameters of the light entering the polarimeter. The detected intensity, $I^{'}$, can be written as a complete orthogonal series:

$$\begin{matrix} I^{'}=I_{DC}+I_{\Omega_{1}}+I_{\Omega_{2}}+I_{\Omega_{mixed}} & (1) \end{matrix}$$

where *I_DC_* denotes the DC signals contributing to the overall intensity,

$$I_{DC}=\frac{1}{2}I+\frac{1}{2}Q \cos^{2}\left( 2\alpha\right)\cos\left( 2\beta\right)+\frac{1}{4}U\sin\left( 4\alpha\right)cos(2\beta)$$

$$+\frac{1}{2}\sin\left( 2\alpha\right)\cos\left( 2\beta\right)\left[ Q\sin\left( 2\alpha\right)-U\cos\left( 2\alpha\right) \right]J_{0}(\delta_{10})$$

$$+\frac{1}{2}\sin\left( 2\alpha\right)\sin\left( 2\beta\right)\left[ Q\cos\left( 2\alpha)+U\sin(2\alpha\right) \right]J_{0}(\delta_{20})$$

$$\begin{matrix} -\frac{1}{2}\cos\left( 2\alpha\right)\sin\left( 2\beta\right)\left[ Q\sin\left( 2\alpha\right)-U\cos\left( 2\alpha\right) \right] J_{0}\left( \delta_{10} \right) J_{0}\left( \delta_{20} \right); & (2) \end{matrix}$$

$I_{\Omega_{1}}$represents the signals associated with the fundamental and higher harmonic frequencies of PEM1,

$$I_{\Omega_{1}}=\left[ \cos\left( 2\alpha\right)\sin\left( 2\beta\right) J_{0} \left( \delta_{20} \right)-\sin\left( 2\alpha\right)\cos\left( 2\beta\right) \right]V \sum_{m=1,3,5\ldots}^{\infty} J_{m}\left( \delta_{10} \right)\sin\left( m\Omega_{1}t \right)$$

$$+\left[ \sin\left( 2\alpha\right)\cos\left( 2\beta\right)-\cos\left( 2\alpha\right)\sin\left( 2\beta\right) J_{0}(\delta_{20}) \right]\left[ Q\sin\left( 2\alpha\right)-U cos(2\alpha) \right]$$

$$\times\begin{matrix} \sum_{n=2,4,6\ldots}^{\infty} J_{n}\left( \delta_{10} \right)\cos\left( n\Omega_{1}t \right); & (3) \end{matrix}$$

$I_{\Omega_{2}}$ denotes the signals associated with the fundamental and higher harmonic frequencies of PEM2,

$$I_{\Omega_{2}}=\sin\left( 2\beta\right)J_{0}\left( \delta_{10} \right)V \sum_{m=1,3,5\ldots}^{\infty} J_{m}\left( \delta_{20} \right) sin(m\Omega_{2}t)$$

$$+\left\{ \sin\left( 2\alpha\right)\sin\left( 2\beta\right)\left[ 1-J_{0}(\delta_{10}) \right]\left[ Q\cos\left( 2\alpha\right)+U\sin\left( 2\alpha\right)+U\sin\left( 2\beta\right) J_{0}(\delta_{10} \right] \right\}$$

$$\times\begin{matrix} \sum_{n=2,4,6\ldots}^{\infty} J_{n}\left( \delta_{20} \right)\cos\left( n\Omega_{2}t \right); & (4) \end{matrix}$$

and, $I_{\Omega_{mixed}}$ represents the terms associated with the frequency differences and frequency sums of both PEMs,

$$I_{\Omega_{mixed}}=\cos\left( 2\alpha\right)\sin\left( 2\beta\right)V\sum_{m=1,3,5\ldots}^{\infty} \sum_{n=2,4,6\ldots}^{\infty} J_{m}\left( \delta_{10} \right) J_{n}\left( \delta_{20} \right)\sin\left[ \left( m\Omega_{1}\pm n\Omega_{2} \right)t \right]$$

$$\pm\sin\left( 2\beta\right)V\sum_{n=2,4,6\ldots}^{\infty} \sum_{m=1,3,5\ldots}^{\infty} J_{n}\left( \delta_{10} \right) J_{m}\left( \delta_{20} \right)sin\left[ \left( n\Omega_{1}\pm{m\Omega}_{2} \right)t \right]$$

$$\mp\sin\left( 2\beta\right)\left[ Q\sin\left( 2\alpha\right)-U\cos\left( 2\alpha\right) \right]$$

$$\times\sum_{m_{1}=1,3,5\ldots}^{\infty} \sum_{m_{2}=1,3,5\ldots}^{\infty} J_{m_{1}}\left( \delta_{10} \right) J_{m_{2}}\left( \delta_{20} \right)cos\left[ \left( m_{1}\Omega_{1}\pm m_{2}\Omega_{2} \right)t \right]$$

$$-\cos\left( 2\alpha\right)sin(2\beta)\left[ Q\sin\left( 2\alpha\right)-U cos(2\alpha) \right]$$

$$\times\sum_{n_{1}=2,4,6\ldots}^{\infty} \begin{matrix} \sum_{n_{2}=2,4,6\ldots}^{\infty} J_{m_{1}}\left( \delta_{10} \right) J_{m_{2}}\left( \delta_{20} \right)\cos\left[ \left( n_{1}\Omega_{1}\pm n_{2}\Omega_{2} \right)t \right]. & (5) \end{matrix}$$

The derivation of Eq.(1)-(5) can be found in our earlier work [1].

A lock-in amplifier measures the signal phase locked to a particular frequency, which may be achieved by multiplying a sinusoidal function of the desired frequency and average over one period [2]. If we set *α* = 45°, *β* = 22.5°, $J_{0}\left( \delta_{10} \right)=J_{0}\left( \delta_{20} \right)=0$, where $\delta_{10}=\delta_{20}\approx2.4048$ rad at the first zero of the Bessel function *J*_0_, and focused on terms related to DC,$\Omega_{1}$,$2\Omega_{1}$ and $2\Omega_{2}$, the relevant expressions are simplified. Arranged in the order of the *I*, *Q*, *U* and *V* to be measured, the DC signal, $S_{DC}$, is now

$$S_{DC}\begin{matrix} =\frac{1}{2}I, & (6) \end{matrix}$$

the AC signal associated with *Q*, $S_{QU1}$, can be written as,

$$\begin{matrix} S_{QU1}=\frac{\sqrt{2}}{2}J_{2}\left( \delta_{10} \right) Q\cos\left( 2\Omega_{1}t \right), & (7) \end{matrix}$$

that associated with *U*, $S_{QU2}$, can be written as,

$$S_{QU2}\begin{matrix} =\frac{\sqrt{2}}{2}J_{2}\left( \delta_{20} \right) U \cos\left( 2\Omega_{2}t \right), & (8) \end{matrix}$$

and that associated with *V*, $S_{V}$, is now

$$S_{V}\begin{matrix} =-\frac{\sqrt{2}}{2}J_{1}\left( \delta_{10} \right)V\sin\left( \Omega_{1}t \right). & (9) \end{matrix}$$

For the sinusoidal demodulation of a lock-in amplifier, the value of *Q*, *U* and *V* may be obtained by multiplying 1, $\cos\left( 2\Omega_{1}t \right)$, $\cos\left( 2\Omega_{2}t \right)$ or $\sin\left( 2\Omega_{1}t \right)$ with Eq. (1) and integrate the equation over one period which provides a factor of ½ for the average of the sinusoidal function squared. We have,

$$\left( \begin{matrix} S_{DC} \\ S_{QU1} \\ S_{QU2} \\ S_{v} \end{matrix} \right)=\left( \begin{matrix} \frac{1}{2} & 0 & 0 & 0 \\ 0 & \frac{\sqrt{2}}{4}J_{2}\left( \delta_{10} \right) & 0 & 0 \\ 0 & 0 & \frac{\sqrt{2}J_{2}\left( \delta_{20} \right)}{4} & 0 \\ 0 & 0 & 0 & -\frac{\sqrt{2}J_{1}\left( \delta_{10} \right)}{4} \end{matrix} \right)\left( \begin{matrix} I \\ Q \\ U \\ V \end{matrix} \right)\begin{matrix} . & (10) \end{matrix}$$

Solving for (*I*, *Q*, *U*, *V*), we obtain:

$$\left( \begin{matrix} I \\ Q \\ U \\ V \end{matrix} \right)=\left( \begin{matrix} 2 & 0 & 0 & 0 \\ 0 & \frac{2\sqrt{2}}{J_{2}\left( \delta_{10} \right)} & 0 & 0 \\ 0 & 0 & \frac{2\sqrt{2}}{J_{2}\left( \delta_{20} \right)} & 0 \\ 0 & 0 & 0 & -\frac{2\sqrt{2}}{J_{1}\left( \delta_{10} \right)} \end{matrix} \right)\left( \begin{matrix} S_{DC} \\ S_{QU1} \\ S_{QU2} \\ S_{v} \end{matrix} \right)\begin{matrix} . & (11) \end{matrix}$$

Analysis of the terms in Eq. (1)-(5) for general *α*, *β* and PEM peak retardation settings shows that the Stokes parameters and the signals recovered are related by the matrix *G* as follows,

$$\left( \begin{matrix} S_{DC} \\ S_{QU1} \\ S_{QU2} \\ S_{v} \end{matrix} \right)\begin{matrix} =G\left( \begin{matrix} I \\ Q \\ U \\ V \end{matrix} \right), & (12) \end{matrix}$$

$$G=\begin{matrix} \left( \begin{matrix} g_{1} & g_{2} & g_{3} & 0 \\ 0 & g_{4} & g_{5} & 0 \\ 0 & g_{6} & g_{7} & 0 \\ 0 & 0 & 0 & g_{8} \end{matrix} \right), & (13) \end{matrix}$$

where *g_i_* (*i* = 1,…,8) are the nonzero elements. Inverting *G* leads to the Stokes parameters being determined from the measured signals:

$$\left( \begin{matrix} I \\ Q \\ U \\ V \end{matrix} \right)\begin{matrix} =K\left( \begin{matrix} S_{DC} \\ S_{QU1} \\ S_{QU2} \\ S_{v} \end{matrix} \right), & (14) \end{matrix}$$

where $K=G^{-1}$:

$$K=\begin{matrix} \left( \begin{matrix} k_{1} & k_{2} & k_{3} & 0 \\ 0 & k_{4} & k_{5} & 0 \\ 0 & k_{6} & k_{7} & 0 \\ 0 & 0 & 0 & k_{8} \end{matrix} \right), & (15) \end{matrix}$$

and where *k_i_* (*i* = 1,…8) are the nonzero elements of *K*. The form of *K* in Eq. (14) is a necessary condition for the *K* to exist as the inversion of *G*. In practice, the nonzero elements *k_i_* (*i* = 1,..8) are determined experimentally by a non-linear least square algorithm with different states of polarisation light in a calibration process [3], which, together with Eq.(15), provides the necessary and sufficient conditions for the existence of *K*.

The discussion above shows demodulation with a sinusoidal function in the lock-in signal recovery, which is applicable for LED implementation. In the prototype microscope implementation with the laser source, the demodulation timing unit (DTU) provides, in effect, square wave demodulation. A square wave can be expanded as a Fourier series with missing even harmonics. Normally, in a lock-in amplifier with square wave demodulation, a bandpass filter is provided to filter out the higher harmonics of reference frequency. However, as an example, if we consider the terms containing *V* associated with PEM (Eq. (3)),

$$\left[ \cos\left( 2\alpha\right)\sin\left( 2\beta\right) J_{0} \left( \delta_{20} \right)-\sin\left( 2\alpha\right)\cos\left( 2\beta\right) \right]V \sum_{m=1,3,5\ldots}^{\infty} J_{m}\left( \delta_{10} \right)\sin\left( m\Omega_{1}t \right),$$

all higher harmonics contribute to the coefficient of *V*, hence, not only it is unnecessary to filter the signals associated with the higher harmonics, but also their contribution improves the sensitivity for the signal associated with *V*.

A new calibration is required, if there is a change to the polarimeter angular settings, the wavelength of the source and the PEM peak retardation settings.

**The relationship between Stokes polarimetric imaging, Mueller matrix imaging and birefringence imaging**

Although the present work focuses on Stokes polarimetric imaging, it may be helpful also to consider its relationship to Mueller matrix and birefringence imaging. The input Stokes parameters $(I,Q,U,V)$ and the output Stokes parameters $(I^{'},Q^{'},U^{'},V^{'})$ at a sample is related by a Mueller matrix *M*,

$$\left( \begin{matrix} I^{'} \\ Q^{'} \\ U^{'} \\ V^{'} \end{matrix} \right)\begin{matrix} =M\left( \begin{matrix} I \\ Q \\ U \\ V \end{matrix} \right), & (16) \end{matrix}$$

where,

$$M=\begin{matrix} \left( \begin{matrix} m_{11} & m_{12} & m_{13} & m_{14} \\ m_{21} & m_{22} & m_{23} & m_{24} \\ m_{31} & m_{32} & m_{33} & m_{34} \\ m_{41} & m_{42} & m_{43} & m_{44} \end{matrix} \right), & (17) \end{matrix}$$

is to be determined at each pixel for Mueller matrix imaging. With a given input state of polarisation, Stokes polarimetric imaging would allow the output Stokes parameters $(I^{'},Q^{'},U^{'},V^{'})$ to be determined at every pixel. So, in principle, a minimum of four suitably chosen input polarisation states are required for the measurements of the four set of corresponding output Stokes parameters for each pixel. The Mueller matrix, *M*, may then be determined by solving 16 simultaneous equations for the 16 matrix elements at each pixel [4].

If a sample is purely birefringent, for the normalised input Stokes parameters, $(1, q, u, v)$, the matrix *M* is simplified to [5]

$$M=\begin{matrix} \left( \begin{matrix} 1 & 0 & 0 & 0 \\ 0 & \cos\left( 4\gamma\right)\sin^{2} \left( \frac{\delta}{2} \right)+\cos^{2}\left( \frac{\delta}{2} \right) & \sin\left( 4\gamma\right)\sin^{2} \left( \frac{\delta}{2} \right) & \sin\left( 2\gamma\right)sin(\delta) \\ 0 & \sin\left( 4\gamma\right)\sin^{2} \left( \frac{\delta}{2} \right) & -cos \left( 4\gamma\right)\sin^{2} \left( \frac{\delta}{2} \right)+\cos^{2}\left( \frac{\delta}{2} \right) & \cos\left( 2\gamma\right)sin(\delta) \\ 0 & -\sin\left( 2\gamma\right)sin(\delta) & \cos\left( 2\delta\right)sin(\delta) & cos(\delta) \end{matrix} \right), & (18) \end{matrix}$$

where *γ* is the orientation angle of the fast axis with respect to the polarimeter *x*- axis and *δ* is the phase retardation between the fast axis and the slow axis. Given an input of a purely circularly polarised light with normalised Stokes parameters (1, 0, 0, 1), polarimetric imaging of the output Stokes parameters would lead to the determination of *δ* through the relationship $v^{'}=\cos\left( \delta\right)$ for each pixel. Further given an input of a purely linearly polarised light with its polarisation plane at 0° with respect to the *x*- axis, (1, 1, 0, 0), *γ* would be determined from the equation: $q^{'}=\left[ \cos\left( 4\gamma\right)\sin^{2}\left( \frac{\delta}{2} \right)+\cos^{2}\left( \frac{\delta}{2} \right) \right]$. Since the measurement of the output Stokes parameters for a given input polarisation state would provide four simultaneous equations, in principle, with a suitably chosen input polarisation state, the two unknowns, namely *γ* and *δ*, may be determined with its corresponding output Stokes parameters at each pixel.

**The LC droplets imaging by a conventional polarised light microscope**

For comparison, the LC droplets of the identical batches were also examined by a transmission light microscope operated either in transmission mode or in cross-polariser mode. A Leica Microsystems Ltd polarised light microscope (model DM 2700M) equipped with a pair of linear polarisers was used for the imaging. Using white light illumination, the images were captured with a Nikon D3000 digital colour camera.


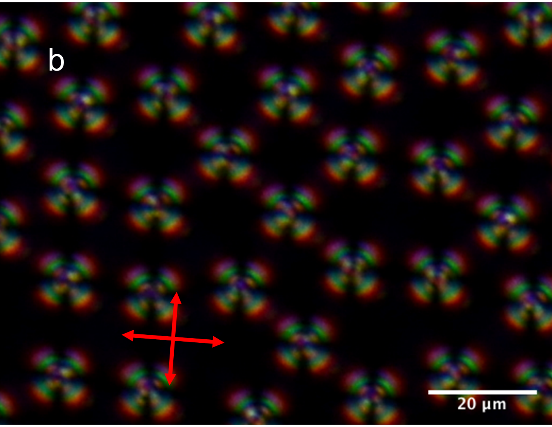

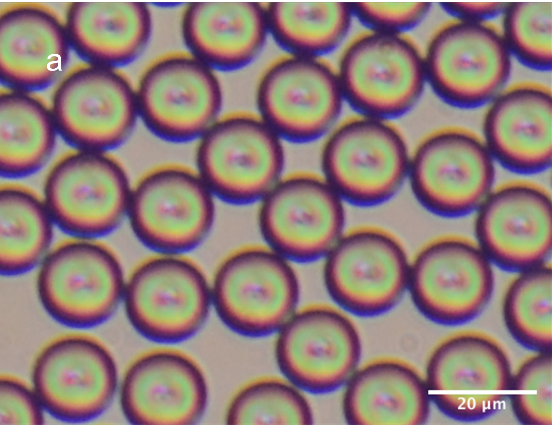

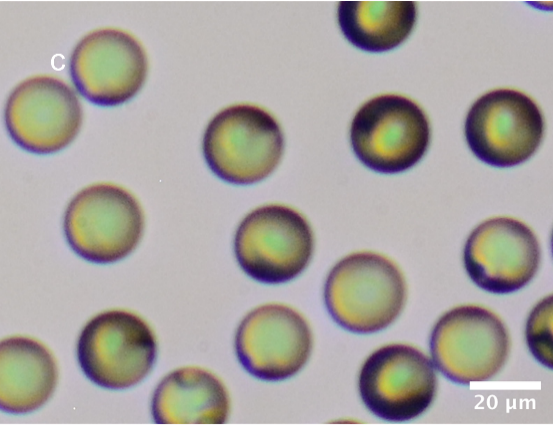

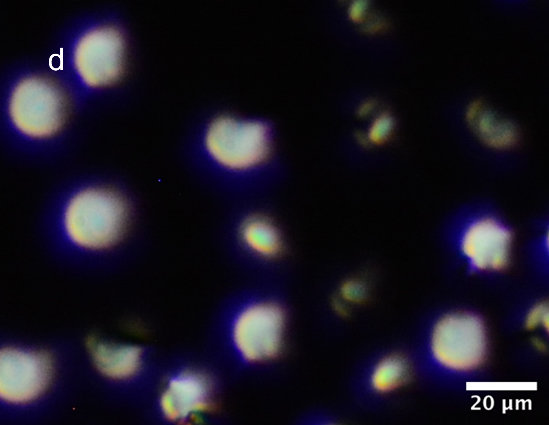

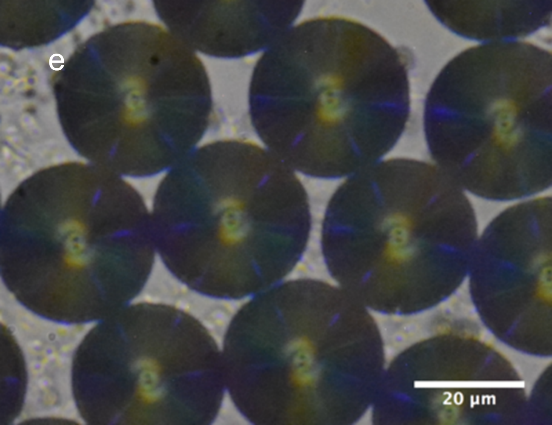

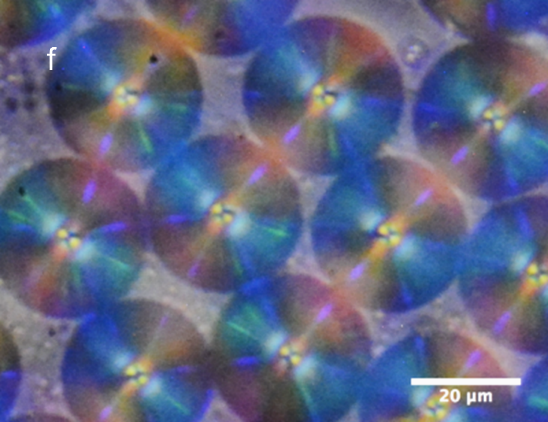


Figure S2. Micrographs of LC droplets: (a) transmission image (b) cross-polariser image of those with the internal structure A; (c) transmission image (d) cross-polariser image, the internal structure B; and (e) transmission image (f) cross-polariser image, the internal structure C. The red arrows in (b) indicate the directions of the cross-polariser arrangement, applicable also to images (d) and (f).

Images of LC droplets of the three distinct structures are shown in Fig S2. Fig S2(a) and (b) are the transmission intensity image and the cross-polariser image, respectively, of radially aligned droplets, E7 LC droplets with a monolayer of mixed lipids at the surface (‘structure A’). Fig S2(c) and (d) are those for droplets of bipolar structures, PVA wrapped E7 droplets having a planar anchor at the boundary (‘structure B’). And, Fig S2(e) and (f) are the images of droplets with a chiral ordering along the radial direction, PVA wrapped E7 droplets doped with a chiral nematic LC twist agent (‘structure C’).

**Reference list:**

[1] Wei, G. Cook, P.J., Jones, G.A. & Shen, T.H. Experimental determination of the Stokes parameters using a dual photoelastic modulator system. *Appl. Optics* **49,** 2644-2652 (2010).

[2] Meade ML. Lock-in amplifier: principles and applications. (P. Peregrinus, 1983).

[3] Cook, P.J. A study of focussed ion beam patterned thin magnetic films with soft x-ray and magneto-optical microscopy, PhD Thesis, University of Salford (UK) 2010.

[4] De Martino, A. et al. Optimized Mueller polarimeter with liquid crystals. Optics Letters 28, 616-618 (2003).

[5] Kliger, D. S. Lewis, J. W. and Randall, C. E. Polarized Light in Optics and

Spectroscopy (Academic, Boston, 1990).
